# Operative stewardship: reclaiming the role of surgical source control in dental antimicrobial stewardship

**DOI:** 10.3389/froh.2026.1868747

**Published:** 2026-06-16

**Authors:** Michael V. Joachim, Alex Abramson

**Affiliations:** 1 Unit of Oral and Maxillofacial Surgery and Department of Plastic Surgery, Shamir (Assaf ha-Rofeh) Medical Center, Affiliated to the Gray Faculty of Medical and Health Sciences, Tel Aviv University, Tzrifin, Israel; 2 Department of Oral and Maxillofacial Surgery, Barzilai Medical Center, Affiliated to the Faculty of Health Sciences, Ben-Gurion University of the Negev, Ashkelon, Israel

**Keywords:** antimicrobial resistance, antimicrobial stewardship, defensive prescribing, dental antibiotic prescribing, maxillofacial surgery, odontogenic infections, surgical source control

## Abstract

Dentistry accounts for approximately 10% of all antibiotic prescriptions globally, with evidence consistently demonstrating that the majority of these prescriptions are inappropriate. Current dental antimicrobial stewardship programs are defined almost exclusively around prescribing behavior—optimizing antibiotic choice, dose, and duration. This pharmacological framing, while necessary, is structurally incomplete. It overlooks the primary clinical intervention that renders antibiotics unnecessary in the first place: surgical source control through incision and drainage or tooth extraction. In established odontogenic abscesses, systemic antibiotics operate in a profoundly hostile environment—avascular, necrotic tissue with acidic pH and pus containing antibiotic inhibitors—rendering antibiotic monotherapy insufficient as a definitive treatment and clinically inadequate as a substitute for operative source control. A growing pattern of defensive prescribing in community dental practice, driven by time pressure, patient expectations, and avoidance of operative procedures, delays definitive treatment and drives infection progression to secondary care. Maxillofacial emergency departments increasingly function as stewards of last resort, performing the drainage procedures that were clinically indicated days prior, at substantially greater cost to patients and health systems. This perspective argues that true antimicrobial stewardship in dentistry must expand its definition to encompass the operative decision itself: when infection is drainable or its source is extractable, operative management is the stewardship act. We propose the concept of Surgical Stewardship and outline its implications for clinical practice, dental education, and the community-to-hospital referral interface.

## 1 Introduction

Antimicrobial resistance is one of the most consequential threats facing global health. In 2019, resistant bacterial infections were directly responsible for approximately 1.27 million deaths worldwide, with an associated burden approaching five million deaths when resistance is considered a contributing factor (1, 2). Without coordinated action, annual mortality from antimicrobial resistance is projected to reach ten million by 2050—surpassing cancer as a cause of death (3).

Dentistry contributes substantially to this global burden. Dentists account for approximately 10% of all outpatient antibiotic prescriptions across healthcare globally (4–6). In the United States alone, dentists wrote over 216 million antibiotic prescriptions between 2012 and 2019, placing the profession among the top three outpatient antibiotic prescribers by volume (7, 8). The volume is compounded by consistent evidence of suboptimality: estimates of non-guideline-concordant dental antibiotic prescribing range from 50% to over 80% across multiple countries and healthcare systems (1, 9, 10).

In response, dental antimicrobial stewardship programs have emerged as a recognized policy priority, aiming to optimize the quantity and quality of antibiotic prescriptions—the right drug, right dose, right duration for the right indication (3, 4). Systematic reviews confirm that this pharmacological orientation is near-universal: education, audit and feedback, guideline dissemination, and prescribing decision support tools (1, 11). What none of them address is operative management as a stewardship domain—the decision to drain an abscess or extract a tooth rather than prescribe. Antibiotics provided in the absence of that decision are not simply unnecessary; they are biologically inadequate, clinically counterproductive, and system-level drivers of escalating hospital burden. We propose the concept of Surgical Stewardship as the conceptual foundation for addressing this gap.

## 2 The biology of failure: why antibiotics cannot replace the scalpel

### 2.1 The abscess as an antibiotic-hostile environment

The failure of antibiotics as monotherapy for established odontogenic abscesses is not a matter of drug selection—it is a consequence of the microenvironment in which those drugs are asked to operate.

Odontogenic infections originate in the endodontic flora of non-vital teeth or the periodontium, and are characteristically polymicrobial, dominated by anaerobic species including *Fusobacterium*, *Prevotella*, *Porphyromonas*, and *Treponema*, alongside facultative streptococci (12, 13). As infection progresses toward abscess formation, aerobic bacteria deplete local oxygen, creating an anaerobic, nutrient-rich environment in which anaerobes proliferate and secrete tissue-destructive toxins, culminating in liquefaction and pus formation (12, 14). The abscess cavity that results is avascular, necrotic, and anaerobic microenvironment is structurally hostile to antibiotic activity through several converging mechanisms.

Systemic antibiotics fail in this environment through several converging mechanisms: the absence of vascular perfusion prevents drug delivery to the site regardless of serum concentration; the acidic pH of the abscess alters ionization of pH-sensitive agents including beta-lactams; the polymicrobial biofilm requires antibiotic concentrations 100–1,000-fold higher than for planktonic bacteria; and pus itself contains antibiotic inhibitors that render agents functionally inactive within the lesion (15, 16).

These mechanisms collectively explain a principle that the surgical and infectious disease literature has stated for decades: antibiotics must never be used as a replacement for appropriate surgical drainage, but only as adjunctive therapy (12, 17). A multi-university expert consensus on the antimicrobial treatment of odontogenic infections is equally direct: antibiotic administration alone is often insufficient to eradicate the infection, and the etiological surgical intervention—drainage or extraction—must be considered as the primary treatment arm (18). This is not to suggest that antibiotics have no role in odontogenic infection management. Where systemic involvement, spreading cellulitis, fever, or immunocompromise is present, antibiotics remain an essential adjunct to operative treatment. The Surgical Stewardship argument concerns specifically the substitution of antibiotics for operative treatment when operative treatment is available and indicated—not the appropriate adjunctive use of antibiotics alongside it. This principle is not new. What is new is the recognition that its systematic violation constitutes a stewardship failure, not merely a clinical one.

### 2.2 The clinical evidence for surgical primacy

The clinical evidence aligns precisely with this biological rationale, and it runs through the highest levels of the dental guidelines’ hierarchy.

The UK Faculty of Dental Surgery and Faculty of General Dental Practice antimicrobial prescribing guidance states that the majority of uncomplicated acute dental infections should be treated by removal of the cause through drainage, removal of infected pulp contents, or extraction—a strong recommendation—and that antibiotics are indicated only as an adjunct in the presence of elevated temperature, systemic spread, or local lymph node involvement (6). A Cochrane review identified within those guidelines, comparing penicillin with placebo as an adjunct to endodontic therapy for adults with localized periapical abscess and necrotic pulp, found no difference in outcomes between groups—including pain and swelling (19). The antibiotic did nothing measurable.

The 2019 American Dental Association clinical practice guideline on antibiotic use for urgent pulpal and periapical conditions issues a strong recommendation against prescribing systemic antibiotics as an adjunct to operative treatment when that treatment is immediately available, for immunocompetent adults with pulp necrosis and localized acute apical abscess (20).

## 3 The community problem: defensive prescribing as a substitute for surgery

### 3.1 The scale of operative avoidance

The gap between the clinical principle of surgical primacy and what occurs in community dental practice is strikingly deep and well-documented. A cross-sectional study of antibiotic prescribing among general dental practitioners in Wales found that antibiotics were prescribed to 57.4% of 568 patients attending with acute dental conditions, and that 70.6% of all antibiotic prescriptions were made without any accompanying operative intervention whatsoever (10). Only 19.0% of prescriptions were in situations where clinical guidelines supported antibiotic use. In other words, the overwhelming majority of dental antibiotic prescriptions were given instead of—not in addition to—operative treatment.

The factors independently associated with antibiotic prescription in the absence of clinical infection tell the structural story directly. Patient unwillingness or inability to accept operative treatment carried an odds ratio of 4.89 for antibiotic prescription; insufficient clinical time to complete operative treatment, 10.21; failure of previous operative treatment, 13.57; and patient request for antibiotics, 3.69 (10). These associations reveal that antibiotic prescribing in primary care dentistry is driven not by the clinical indication, but by the avoidance of operative intervention.

The pattern is consistent across countries and settings. Dental antibiotic prescribing rates in the United States remained unchanged between 2012 and 2019 despite national prescribing reductions and guideline updates (7). Cross-national comparisons show the United States prescribed twice the dental antibiotic rate of Australia, and a global scoping review confirms substantial suboptimal prescribing across high-, middle-, and low-income country contexts alike (5, 21). It is a behavioral and structural problem, not a knowledge gap.

The consistency of this pattern across high-income healthcare systems reinforces its structural character. In the United States, dental antibiotic prescribing rates remained essentially unchanged between 2012 and 2019, a period during which national stewardship campaigns and updated clinical guidelines were widely disseminated—indicating that knowledge alone does not drive the reduction in prescribing volume (7). Cross-national comparison across four jurisdictions—Australia, England, the United States, and British Columbia—found the United States prescribed at more than twice the dental antibiotic rate of Australia, a disparity that persists after controlling for population size and that aligns with documented differences in operative capability and appointment structures between systems (21). At a global level, a scoping review of antibiotic use across dental practices in multiple countries confirms that substantial suboptimal prescribing is present across high-, middle-, and low-income settings alike, and explicitly attributes the spike in prescribing during the COVID-19 pandemic to the reduction in routine operative treatments—a natural experiment in which operative avoidance directly produced increased prescribing volume (5). A systematic review of dental antibiotic stewardship interventions found that 70.6% of antibiotics in the UK were prescribed without any accompanying active dental treatment, and that more than 80% of antibiotic prophylaxis prescriptions in the United States were deemed unnecessary—figures that have remained largely stable across studies conducted in different healthcare contexts and time periods (1).

### 3.2 The behavioral drivers: defensive prescribing

It is important to state at the outset that the barriers to operative management in community dental practice are real, well-documented, and in many cases not of the practitioner's making: limited emergency appointment time, the genuine difficulty of achieving adequate local anesthesia in infected and acidotic tissue, patient refusal of immediate surgery, restricted referral access, socioeconomic constraints on patient attendance, and medico-legal pressures that render prescribing a perceived lower-risk act than operative intervention or urgent referral. Qualitative research makes the mechanisms visible in a way that audit data cannot. Dentists in multiple settings describe using antibiotics deliberately as a substitute for operative treatment they cannot or will not provide at that encounter. Time pressure during emergency appointments, patient refusal of immediate surgery, inability to follow up, and fear of clinical complications all feature in these accounts (22). The use of antibiotics to get patients “through to Monday,” to manage patients until specialist referral is possible, or to cover situations where definitive treatment is unaffordable or logistically inaccessible, represents a pattern described consistently across the UK, Canada, Germany, and Jordan (9, 22, 23). Dentists who engage in this practice frequently acknowledge its clinical inadequacy—they know the antibiotic is not the correct treatment—but weigh the risks of operative avoidance as acceptable relative to the immediate clinical and social pressures they face (22). Fear of legal consequence reinforces this pattern: prescribing an antibiotic is perceived as a defensible act, whereas the decision to refer for urgent drainage carries a different risk profile (9).

The downstream consequence of this behavioral pattern is predictable. Failed or incomplete operative management does not resolve infection—it postpones the clinical reckoning while the infection progresses. Recognizing this pattern as a systemic failure is not an indictment of individual practitioners operating under genuine constraints—it is an argument that those constraints are stewardship problems that the system, not the practitioner alone, must address.

### 3.3 The hot tooth: where avoidance is most consequential

The symptomatic necrotic tooth with acute apical abscess—the clinical scenario colloquially known as the hot tooth—represents the context in which operative avoidance is simultaneously most common and most damaging. The combination of acute pain, patient distress, and the well-recognized difficulty of achieving adequate local anaesthesia in infected, acidotic tissue creates a genuine and understood clinical barrier (24). Practitioners confronted with this scenario may prescribe antibiotics to reduce inflammation before rescheduling operative treatment—a clinically rational-feeling but pharmacologically inadequate response (19).

The fundamental problem is that the hot tooth is precisely the case where antibiotics are least effective. The necrotic, avascular pulp and the forming periapical abscess represent the most antibiotic-hostile tissues in the body. The ADA guideline goes further: even for symptomatic irreversible pulpitis—a condition involving vital, inflamed pulp tissue, not yet necrotic—systemic antibiotics provide no meaningful clinical benefit and are associated with large harms (13, 20). If antibiotics cannot reduce pulpitis pain or swelling in vital pulps, they are far less likely to resolve the bacterially-loaded, avascular, pus-filled cavity of an established abscess. In the established abscess, every prescription written instead of operative management is, at best, a temporary and incomplete response—one that does not resolve the microbial source and carries a clinically meaningful risk of allowing the infection to progress along fascial planes.

### 3.4 Resistance consequences of operative avoidance

The antimicrobial resistance consequences of dental overprescribing are not theoretical—they are measurable, agent-specific, and in at least one domain reversible. Understanding them is essential to the Surgical Stewardship argument, because it reframes each episode of defensive prescribing in lieu of operative intervention not merely as a clinical missed opportunity but as a direct contribution to the resistance environment that will eventually reduce the effectiveness of antibiotics for all patients.

Resistance rates among oral pathogens causing odontogenic infections are already clinically significant. In Germany—one of the most studied dental prescribing contexts in Europe—dentists were the second-largest group of antibiotic prescribers in 2024, issuing prescriptions to 2.3 million patients nationally. Despite national guidelines recommending aminopenicillins as first-line therapy and reserving clindamycin for patients with confirmed penicillin allergy, clindamycin continues to be frequently prescribed. This is clinically consequential: resistance rates of 17%–19% have been documented among clinically relevant oral pathogens including Streptococcus spp. and Staphylococcus aureus for this agent, rates that are high enough to materially undermine empirical clindamycin therapy for odontogenic infections (2, 25).

Critically, this resistance is not fixed. Evidence from Finland demonstrates that the relationship between prescribing volume and resistance rates operates in both directions. Following nationwide recommendations to reduce macrolide antibiotic prescribing in outpatient settings, macrolide consumption in Finland fell from 2.40 to 1.38 defined daily doses per 1,000 inhabitants per day between 1991 and 1992. This was followed by a steady and measurable decline in erythromycin resistance among group A streptococcal isolates: from 16.5% in 1992 to 8.6% in 1996 (26). The dental corollary is precise: dentists can both generate and reduce resistance at a population level through their prescribing behaviour. Each course of amoxicillin or clindamycin prescribed instead of a drainage or extraction is not pharmacologically neutral—it exerts selective pressure on the oral microbiome and the broader community reservoir of resistance genes.

Beyond resistance in oral pathogens, dental antibiotic prescribing carries a specific, mechanistically understood adverse outcome that extends beyond the oral cavity. Broad-spectrum antibiotic courses—including amoxicillin and clindamycin, the two most common dental agents—disrupt the enteric flora and are independently associated with Clostridioides difficile colonization and infection (27). This is not a remote or theoretical risk: C. difficile infection carries significant morbidity, a recurrence rate of 20%–30%, and—in elderly or immunocompromised patients—meaningful mortality. A dental antibiotic prescribed in lieu of a 10-min extraction is not merely inadequate treatment for the abscess; it is a course of systemic antibiotic therapy with all the resistance and enteric consequences that entails. The Surgical Stewardship model, by making operative source control the primary act and reserving antibiotics for cases where the clinical indication is unambiguous, eliminates this adverse exposure at source.

## 4 The hospital end: maxillofacial emergency departments as stewards of last resort

### 4.1 The escalation pathway

When operative intervention is avoided in primary care, odontogenic infections do not remain localized. The anatomy of the head and neck provides a system of connected fascial planes through which infection spreads with predictable and potentially lethal efficiency: from a periapical abscess manageable in any dental practice, to masticator and submandibular space involvement, thence to the parapharyngeal, retropharyngeal, and carotid spaces, and ultimately to the mediastinum—infections at this stage requiring thoracic surgery with significant morbidity and mortality (28). This anatomical reality has been recognized for decades: infections in adults have become more severe over time, spreading more rapidly through fascial planes and requiring tracheotomy with increasing frequency, and late referral makes their management substantially more precarious (12, 29).

Established criteria for hospital referral—rapidly progressive cellulitis, dysphagia, trismus, spread to deep facial spaces, fever, systemic compromise, or failure of initial treatment—define the threshold at which community management is no longer safe (6, 18, 24, 29). Each such case represents a point at which earlier operative intervention could have altered the trajectory.

### 4.2 The hospital burden: quantified consequences

**Figure 1:**
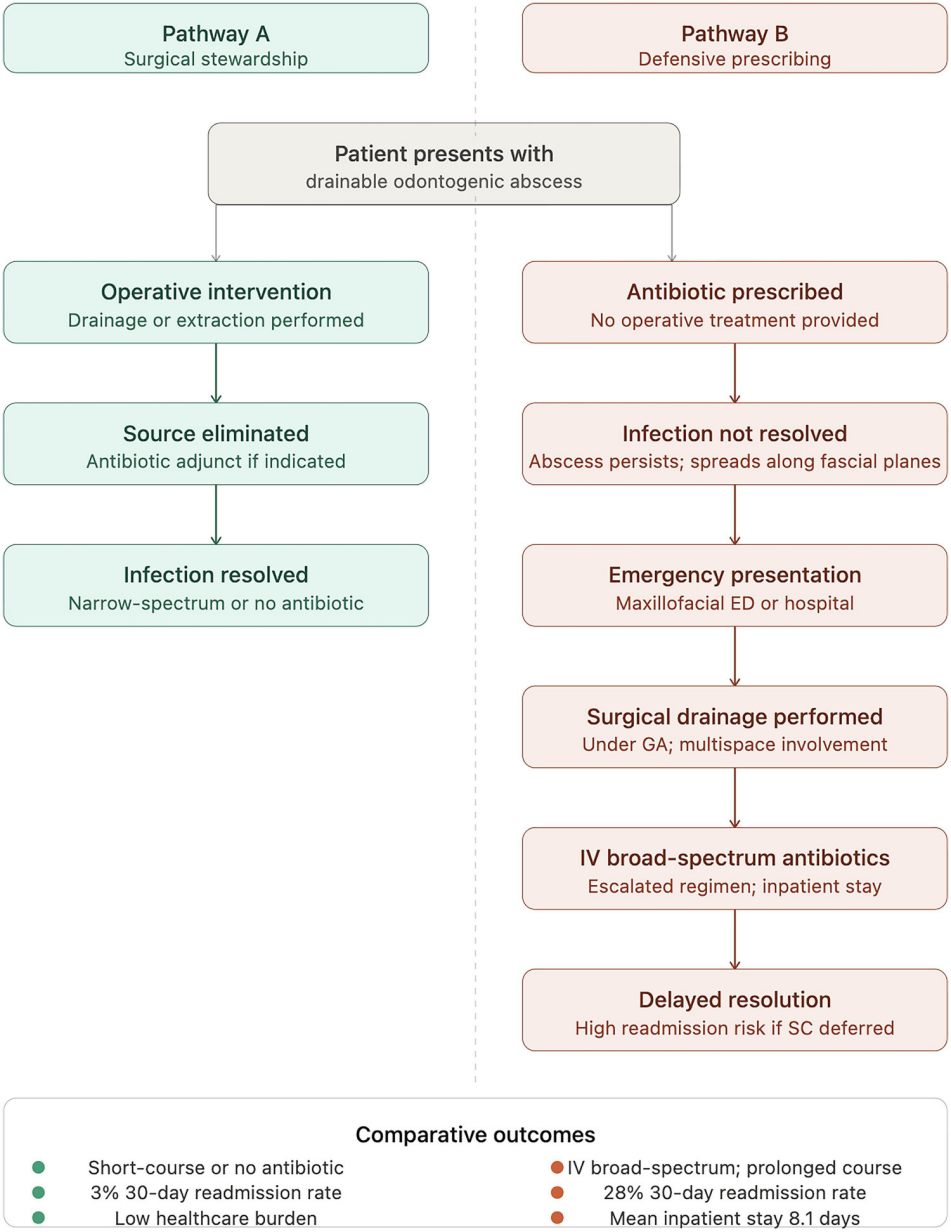
Two clinical pathways for the management of drainable odontogenic abscess in community dental practice. Pathway A (Surgical Stewardship) represents operative management at first contact, resulting in source elimination, short-course or no antibiotic use, and infection resolution. Pathway B (Defensive Prescribing) represents antibiotic substitution without operative treatment, leading to infection progression along fascial planes, emergency hospital presentation, surgical drainage under general anaesthesia, escalation to intravenous broad-spectrum antibiotics, and prolonged inpatient stay. GA, general anaesthesia; IV, intravenous; ED, emergency department; SC, surgical source control.

The consequence of failing to provide surgical source control—whether that failure occurs in community primary care or at the point of hospital presentation—is now directly supported by outcome data from inpatient and emergency department settings. A retrospective cohort study of 78 patients presenting with odontogenic infection to a large urban safety-net hospital in Detroit demonstrated a strong association between definitive surgical source control—extraction and/or abscess drainage—and 30-day all-cause readmission: patients who received source control had a readmission rate of 3.3% compared with 27.8% in those who did not (*p* = 0.001), representing an 88% relative risk reduction. As a retrospective observational study, residual confounding cannot be excluded, though the groups did not differ significantly in demographic or clinical characteristics (30). Among admitted patients whose source control was deferred to outpatient follow-up, 87% were subsequently lost to follow-up—meaning that the definitive surgical treatment was never delivered, and the cycle of antibiotic prescription without resolution continued (30). Figure 1 illustrates the two clinical pathways and their divergent outcomes at the system level.

A separate analysis at a safety-net hospital in San Antonio reported similarly: readmission rates of 28% in patients without surgical source control versus 3% in those who received it (*p* = 0.001), consistent with a pattern in which infections receiving antibiotics without source control remain unresolved and subsequently require the operative management that was deferred at first presentation (31).

The inpatient resource implications are substantial. A retrospective analysis of patients admitted with severe odontogenic infections found a mean hospital stay of 8.1 days, with significantly longer stays in patients with systemic comorbidities, and concluded that inpatient treatment of severe odontogenic infections carries high costs and “can create important limitations in bed occupation rates, budget expenditure and staff allocation” (32). These data quantify, in operational terms, the downstream cost of the community operative avoidance described in Section 3.

The most direct available evidence linking community-level operative avoidance to downstream prescription burden comes not from a cohort study but from a natural experiment. During the COVID-19 restrictions of March to June 2020, NHS England directed dental practices to manage patients remotely using the advice-analgesia-antibiotic (AAA) approach, suspending face-to-face operative care. Dental antibiotic prescribing across England was 25% higher in April to July 2020 compared with the same period in 2019, against a pre-pandemic background of year-on-year decline; the increase peaked in June 2020 at 28% above the April rate, with London recording a 60% increase (33). The UK Parliament's Health and Social Care Committee documented concurrent patient harm from this approach directly: patients were remotely prescribed antibiotics but returned with pain or further swelling because the source of their dental problem had not been operatively addressed (33). This pattern—operative avoidance generating antibiotic prescribing, which fails to resolve the infection, which generates re-presentation—is precisely the community-to-downstream escalation pathway that the Surgical Stewardship framework is designed to interrupt. The same pattern was replicated internationally: a global scoping review documented the COVID-era increase in dental antibiotic prescribing across multiple countries, attributing it explicitly to the reduction in routine operative treatments (5).

An important qualification to this evidence must be stated explicitly. A large UK primary care study linking antibiotic prescribing rates to hospital admission outcomes across 19.6 million GP consultations found that practices with higher antibiotic prescribing rates had lower rates of infection-related hospital admissions, with the authors concluding that indiscriminate reductions in antibiotic prescribing may cause harm (34). This finding does not contradict the Surgical Stewardship argument—but it sharpens the precision with which it must be stated. The data apply to a broad range of common infections—respiratory tract infections, urinary tract infections, otitis media—in which antibiotic therapy is often the primary and sometimes the only available management intervention. The Surgical Stewardship framework applies specifically to odontogenic infections in which a drainable source exists: in these cases, operative intervention—incision and drainage, extraction, or pulp extirpation—eliminates the microbial reservoir entirely, removing the clinical indication for antibiotic therapy rather than simply withholding it. The distinction is between operative substitution (treat operatively instead of pharmacologically) and indiscriminate prescribing reduction (prescribe less without providing an alternative). The harm signal identified by that study belongs to the latter; Surgical Stewardship proposes only the former. A prospective longitudinal study directly tracking individual patients from a community dental antibiotic prescription through to hospital presentation for the same infection—establishing the community-to-hospital pathway at patient level, and distinguishing operative-substitution pathways from bare prescribing reduction—remains an important gap in the evidence base.

### 4.3 The antibiotic escalation paradox

The hospital burden of delayed community operative management extends beyond inpatient days and readmission rates. When patients arrive at a Maxillofacial Emergency Department with a progressed odontogenic infection, the antibiotic regimen escalates systematically. The oral community antibiotic—most commonly amoxicillin or amoxicillin-clavulanate—is superseded by intravenous broad-spectrum agents, with review of step-down to oral therapy at 24–72 h post-surgery (6). In Ludwig's angina and other severe multispace infections, intravenous corticosteroids, fluid resuscitation, intensive nursing, and potentially airway intervention are added to the management burden (28).

This is the antibiotic escalation paradox: the community dentist who prescribes an oral antibiotic instead of operating has not avoided antibiotic exposure for the patient or the system—they have deferred it, at greater quantity, broader spectrum, and exponentially greater cost, to the hospital. The Maxillofacial Emergency Department does not simply perform the drainage that the community failed to provide; it corrects the entire pharmacological failure of the primary care encounter. Where operative management in primary care was the appropriate and available response, this pattern—escalating from narrow-spectrum oral agents to broad-spectrum intravenous regimens—represents precisely what antibiotic stewardship programs are designed to prevent (3, 4). When the unit of analysis is the entire care episode rather than the single community prescription, the stewardship failure is far larger than any prescribing audit would capture.

## 5 Toward surgical stewardship: a framework proposal

### 5.1 Redefining the stewardship act

Antimicrobial stewardship, as currently operationalized, treats the prescription as the unit of analysis—asking whether the right drug was chosen at the right dose for the right duration. This is a necessary question, but it is the second question. The first—should an antibiotic have been prescribed at all or should operative treatment have been provided instead?—sits entirely outside the current framework. Surgical Stewardship relocates the primary stewardship decision to the clinical assessment itself: when a drainable abscess is present, drainage is the stewardship act; when an infected tooth can be extracted, extraction is the stewardship act; when pulpal drainage can be established, that is the stewardship act.

For the purposes of this framework, a drainable abscess is defined operationally as a localized, fluctuant collection with clinical evidence of pus formation—an abscess in the established sense—accessible via intraoral incision and drainage or tooth extraction under local anaesthesia within the primary care setting. This stands in contrast to the earlier, diffuse stage of cellulitis, in which infection remains aerobic and no discrete pus cavity yet exists: cellulitis is characterised by diffuse, indurated, rapidly progressing swelling with borders that are enlarging and without fluctuance, whereas abscess is characterised by a localized, smaller, softening collection of anaerobic origin in which the size is diminishing and pus is present (29). When an abscess is accessible, drainage eliminates the source; when only cellulitis is present, surgical drainage cannot be performed and antibiotics, together with urgent operative source removal where feasible, remain the appropriate management (24, 27). A drainable abscess ceases to be manageable in primary care—and therefore falls outside the Surgical Stewardship primary care decision—when it involves moderate- or high-severity anatomic spaces, compromises the airway or vital structures, requires general anaesthesia for access, or is accompanied by systemic compromise including dysphagia, trismus beyond the masticator space, fever, or clinical signs of sepsis: these constitute established criteria for secondary care referral (24, 29).

In each case, the operative intervention eliminates the microbial source and removes the indication for antibiotic therapy entirely. The most powerful tool for reducing inappropriate prescribing is not a better prescribing algorithm—it is operative competence applied at the right moment.

The clinical foundations of this position are longstanding. Sandor et al. state it explicitly (12). Bascones et al. embed surgical treatment as the first of three treatment arms (18). Lockhart et al. and Royal College of Surgeons of England both make operative treatment the primary recommendation, with antibiotics contingent on its availability (6, 20). What Surgical Stewardship adds is the deliberate incorporation of this clinical principle into the definition, measurement, and evaluation of stewardship itself—recognizing that a practitioner who drains an abscess and prescribes no antibiotic has made a stewardship decision of equal or greater importance than a practitioner who selects amoxicillin over clindamycin. To be clear about where the novelty of this proposal lies: source control is not a new concept in infection management—it is a foundational principle of surgical infectious disease, present in the dental and maxillofacial literature for decades. What is new is its deliberate and explicit incorporation into the definition, measurement, and governance of antimicrobial stewardship as a field. Surgical Stewardship does not claim to introduce operative management—it claims that operative management should be counted, tracked, and evaluated as a stewardship act, with the same institutional weight that is currently given to prescribing metrics, audit tools, and educational frameworks.

### 5.2 Implications for community clinical practice

Adopting Surgical Stewardship in community dental practice requires reframing operative capability as a stewardship competency, not merely a clinical one. Current audit tools and stewardship metrics track prescribing outcomes exclusively: number of prescriptions, antibiotic class, treatment duration (1, 3). Under a Surgical Stewardship model, audit would additionally capture operative intervention rates at urgent dental appointments—what proportion of patients presenting with drainable abscesses received drainage, and what proportion received a prescription without operative treatment.

The international consensus core outcome set for dental antibiotic stewardship (COS-DABS) defines the minimum outcomes that studies of stewardship interventions should report: these encompass the quantity of antibiotic prescribing, the appropriateness (quality) of prescribing relative to a defined clinical standard, adverse or poor outcomes including complications arising from disease progression, and a patient-reported outcome relating to normalcy of function (3). Surgical Stewardship proposes one additional operative metric to sit alongside these: the operative intervention rate at urgent dental appointments, defined as the proportion of patients presenting with a localized fluctuant abscess who received surgical source control—drainage or extraction—at first contact, expressed as a percentage of all such presentations. An adequate benchmark would constitute a rate of operative intervention approaching 100% in patients who meet the clinical criteria above, with antibiotic prescription without operative treatment reserved for cases where immediate treatment is clinically or logistically contraindicated. This metric directly captures the stewardship decision that the COS-DABS framework, by focusing exclusively on prescribing outcomes, does not currently address.

Crucially, this operative metric is derivable from data that most dental practice management systems already collect. Appointment type coding, treatment codes for incision and drainage, extraction, and pulp extirpation, and prescription records are routinely captured in NHS and equivalent primary care systems internationally. Linking prescription records to treatment codes at the same appointment—asking whether an antibiotic was issued at an urgent appointment where no operative treatment code was also recorded—would operationalize this metric without requiring new data collection infrastructure. The feasibility of integrating this linkage into existing prescribing audit workflows, such as those used in the NHS Business Services Authority antibiotic prescribing dashboards, represents a practical next step for translating Surgical Stewardship from a conceptual framework into a measurable one.

This reframing also requires acknowledging the barriers that make operative avoidance understandable without accepting them as justifications. Achieving adequate anaesthesia in acutely infected, acidotic tissue is technically demanding (24). Emergency appointment slots in high-volume practices are often inadequate for incision and drainage procedures. Confidence in drainage procedures varies considerably across the general dental practitioner workforce. Patient refusal of immediate operative treatment is a reality (10, 22). A Surgical Stewardship framework names these as stewardship problems requiring stewardship solutions: training in anesthetic technique for infected fields as a stewardship-relevant skill; appointment structures that accommodate urgent operative procedures; referral pathways that prioritize timely surgical access when the community practitioner is unable to operate.

### 5.3 Implications for dental education

Dental curricula currently incorporate antimicrobial stewardship primarily within pharmacology teaching, positioning it as a prescribing competency (9). Knowledge gaps among graduating dental students regarding antibiotic prescribing guidelines are well-documented, and the absence of curricular standardization is identified as a primary driver of outdated prescribing habits that persist into practice (9, 35). Addressing these gaps through pharmacological teaching alone, however, will not resolve a problem that is fundamentally operative in nature.

Surgical Stewardship requires that curricular reform extend from pharmacology into clinical decision-making teaching. Students should learn, explicitly and early, that the operative decision—to drain, extract, or establish pulpal drainage—is an antimicrobial stewardship decision with quantifiable consequences for antibiotic use, infection resolution, hospital burden, and resistance selection. The biological rationale for antibiotic failure in avascular abscess cavities, the documented patterns of defensive prescribing and their drivers, and the hospital outcome data for delayed source control together constitute a coherent and teachable argument for operative primacy. Teaching this argument—rather than merely teaching antibiotic prescribing guidelines—is the generational shift that the field requires (9).

### 5.4 Implications for the community-to-hospital interface

The most systemically significant implication of Surgical Stewardship is for how the relationship between primary care and hospital maxillofacial services is structured. The current model treats hospital referral for drainage as a measure of last resort—an escalation that follows failed antibiotic treatment. Surgical Stewardship inverts this: when drainage is the correct treatment and cannot be provided in primary care, referral for drainage is the first-line response, not the fallback.

Hospital outcome data demonstrate that this failure is not confined to primary care—even within admitted patient populations, source control is frequently deferred, with the majority of those patients lost to follow-up and no definitive treatment ever delivered (30). Rapid referral pathways that function as formal stewardship pathways—not emergency escalation of last resort—are a necessary structural component of any system-level Surgical Stewardship framework.

## 6 Limitations

Several limitations of this perspective article should be acknowledged explicitly. First, the behavioral mechanism linking defensive prescribing to operative avoidance is derived from a small number of qualitative studies, cross-sectional audits, and practitioner surveys; while these are internally consistent and theoretically coherent, they do not constitute a causal evidence base, and it remains possible that operative avoidance reflects structural barriers—appointment duration, anaesthetic failure, patient refusal—rather than a prescribing-first behavioral preference (1, 10).

Second, the Surgical Stewardship framework proposed here has not been empirically tested. Its effect on antibiotic prescribing rates, patient clinical outcomes, infection progression, and downstream hospital burden remains to be demonstrated in prospective or interventional study designs; the framework should therefore be understood as a theoretically grounded clinical proposal requiring empirical development, not a practice-ready intervention protocol.

Third, the audit metric proposed—the operative intervention rate at urgent dental appointments—has not yet been operationalized or validated as a stewardship outcome measure. While it is derivable from existing appointment coding and prescription data in NHS-type systems without requiring new infrastructure, its feasibility, reliability, and sensitivity to change in real-world audit settings would need to be established prospectively before it could function as a reportable quality indicator.

Fourth, the evidence linking community-level operative avoidance to hospital escalation is inferential rather than directly demonstrated by longitudinal patient-level cohort data. The hospital outcome data cited—demonstrating 88% relative risk reduction in 30-day readmission with surgical source control—establish consequences of withholding operative treatment within hospital and emergency department settings (30, 31). The COVID-19 natural experiment provides population-level evidence that operative withdrawal increases prescribing volume and produces unresolved infections (33). However, a prospective study tracking individual patients from a community dental antibiotic prescription through to hospital presentation for the same infection has not been conducted. Furthermore, evidence from general primary care demonstrates that indiscriminate reductions in antibiotic prescribing—without operative substitution—are associated with increased infection-related hospital admissions, underscoring that the Surgical Stewardship proposal must be understood precisely as operative substitution and not as bare prescribing reduction (34).

These limitations do not invalidate the conceptual argument for Surgical Stewardship, which rests on established clinical principles of infection management and on convergent evidence from multiple settings. They do, however, define a clear research agenda: prospective observational studies linking community prescribing events to downstream outcomes; interventional trials testing whether Surgical Stewardship training or structural changes alter operative intervention rates and prescribing; and operationalization of the proposed audit metric in real-world NHS coding environments.

## 7 Conclusion

The dental antimicrobial stewardship field has made genuine progress. The scale of inappropriate prescribing is now well-documented across multiple countries and healthcare systems, the behavioral drivers of overprescribing are increasingly understood, and a growing body of intervention studies demonstrates that prescribing behavior can be changed. This progress should be recognized and built upon.

But the framework has a structural gap. By defining stewardship entirely at the level of the prescription, it leaves unaddressed the clinical decision that—when correctly made—eliminates the need for that prescription. A dentist who drains an abscess and prescribes nothing where no systemic involvement is present is practicing stewardship in its most direct form. A dentist who prescribes amoxicillin as a substitute for draining that abscess—when drainage is immediately available and indicated—has missed the primary stewardship opportunity, regardless of whether the drug chosen was guideline-concordant.

The consequences of this gap are visible in every maxillofacial emergency department that treats patients presenting with multispace odontogenic infections after multiple courses of community antibiotics. These patients did not fail antibiotic treatment. Antibiotic treatment failed them—because it was deployed as a substitute for surgery, in an environment where it was biologically unable to succeed, with predictable and measurable consequences for the patients, the hospitals, and the integrity of antimicrobial agents that all of medicine depends upon.

Surgical Stewardship does not require new drugs, new technologies, or new research. It requires a conceptual reorientation—one that places the operative decision at the center of stewardship thinking, recognizes drainage and extraction as stewardship acts, and builds clinical practice, education, and audit systems around that recognition. The scalpel, in the right hands at the right moment, remains the most effective antimicrobial stewardship instrument available in dentistry.

## Data Availability

The original contributions presented in the study are included in the article/Supplementary Material, further inquiries can be directed to the corresponding author.
